# Causes of misdiagnoses by thyroid fine-needle aspiration cytology (FNAC): our experience and a systematic review

**DOI:** 10.1186/s13000-019-0924-z

**Published:** 2020-01-03

**Authors:** Yanli Zhu, Yuntao Song, Guohui Xu, Zhihui Fan, Wenhao Ren

**Affiliations:** 1 Key laboratory of Carcinogenesis and Translational Research (Ministry of Education), Department of Pathology, Peking University Cancer Hospital and Institute, Beijing, 100142 China; 2 Key laboratory of Carcinogenesis and Translational Research (Ministry of Education), Department of Head and Neck Surgery, Peking University Cancer Hospital and Institute, Beijing, 100142 China; 3 Key laboratory of Carcinogenesis and Translational Research (Ministry of Education), Department of Ultrasound, Peking University Cancer Hospital and Institute, Beijing, 100142 China

**Keywords:** Thyroid, Fine-needle aspiration cytology (FNAC), False-negative, False-positive, Noninvasive follicular thyroid neoplasm with papillary-like nuclear features (NIFTP)

## Abstract

**Objective:**

FNA is a simple, safe, cost-effective and accurate diagnostic tool for the initial screening of patients with thyroid nodules. The aims of this study were to determine the diagnostic utility of FNAC performed in our institution, assess the cytomorphologic features that contribute to diagnostic errors and propose improvement measures.

**Methods:**

A total of 2781 FNACs were included in the study, and 1122 cases were compared with their histological diagnoses. We retrospectively reexamined our discordant (both false-negative and false-positive) cases and performed a systematic review of previous studies on causes of misdiagnoses.

**Results:**

When DC V and DC VI were both considered cytologic-positive, the sensitivity, specificity, positive predictive value (PPV), negative predictive value (NPV) and diagnostic accuracy were 98.3, 30.9, 94.9, 58.3 and 93.5%, respectively. If DC VI was considered cytologic-positive, the sensitivity, specificity, PPV, NPV and diagnostic accuracy of FNAC were 98.0, 84.0, 99.4, 58.3, and 97.5% respectively. The main cause of false-negative diagnoses was sampling error (13/15, 86.7%), while interpretation error led to the majority of the false-positive diagnoses (38/47, 80.9%). Overlapping cytological features in adenomatous hyperplasia, thyroiditis and cystic lesions were the major factors contributing to interpretation errors, while the size and number of nodules may have led to false-negative diagnoses because of heterogeneity and unsampled areas.

**Conclusions:**

The sensitivity and PPV of thyroid FNAC in our institution were higher than those in the published data, while the specificity and NPV were lower. Regarding the FNA category DC V, a frozen section analysis during diagnostic lobectomy is necessary. Multiple passes should be performed in various parts of a large nodule or from different nodules to reduce the risk of false-negative findings. Cytopathologists should strengthen their criteria for the identification of adenomatous hyperplasia, thyroiditis and cystic lesions to avoid false-positive diagnoses. NIFTP has little effect on diagnostic accuracy and the distribution of diagnostic errors.

## Introduction

Thyroid nodules are common; most nodules are benign, and approximately 5% of excised nodules are malignant [1]. The main goal of thyroid fine-needle aspiration (FNA) is to identify the nodules that require surgery and decrease the overall incidence of thyroidectomy among patients with benign disease. Fine-needle aspiration cytology (FNAC) is an efficient and reliable means for the evaluation of thyroid nodules and is considered the gold standard for preoperative diagnoses [2–4]. At the same time, FNA of thyroid nodules has limitations in that both false-negative and false-positive results can occur [5, 6].

The current study was undertaken to determine the diagnostic utility of FNAC performed in our institution by correlating FNAC results with histological diagnoses. We aimed to retrospectively re-examine our discordant (both false-negative and false-positive) cases, to perform a systematic review of previous studies to assess the cytomorphologic features that contributed to diagnostic errors and to propose improvement measures.

Moreover, the new pathologic entity “noninvasive follicular thyroid neoplasm with papillary-like nuclear features” (NIFTP) has been introduced, but whether this procedure will affect the distribution of diagnostic errors remains uncertain.

## Materials and methods

### Thyroid FNA cases

Patients who underwent preoperative thyroid FNA between April 2014 and March 2019 at Peking University Cancer Hospital were identified, and their FNA results were compared with surgical pathology findings. The data were retrieved from the electronic medical records. FNAs were performed by surgeons or sonographers either by palpation or via ultrasound guidance without onsite evaluation. The aspirates were prepared as direct smears (hematoxylin-eosin stain) and/or liquid-based cytology (Papanicolaou stain). All cases were initially classified according to the recommended six diagnostic categories (DCs) of the Bethesda system for reporting thyroid cytopathology (TBSRTC), including nondiagnostic or unsatisfactory (ND/UNS; I); benign (B; II); atypia of undetermined significance or follicular lesion of undetermined significance (AUS/FLUS; III); suspicious for follicular neoplasm or follicular neoplasm (SFN/FN; IV); suspicious for malignancy (SM; V); and malignant (M; VI). Adequacy was determined on the basis of the standard Bethesda criteria [7].

### The false-negative and false-positive diagnoses

When both DC V and DC VI were considered cytologic-positive (both need surgical excision according to the American Thyroid Association guidelines) [8] and when DC II was considered cytologic-negative, a false-negative diagnosis was defined as a nodule that was benign by FNAC (DC II of TBSRTC) and malignant by final histological examination, and a false-positive diagnosis was defined as a nodule with malignant cytology (DC V and DC VI) and postsurgical histological findings of a nonneoplastic lesion or benign neoplasm.

When indeterminant and nondiagnostic cytological findings were not present, DC VI was considered cytologic-positive, and DC II was considered cytologic-negative, a false-negative diagnosis was defined as a nodule that was benign by FNAC (category II of TBSRTC) and malignant by final histological examination, and a false-positive diagnosis was defined as a case that was positive for DC VI by FNA and had postsurgical histological findings of a nonneoplastic lesion or benign neoplasm.

We divided the misdiagnoses into two categories: ‘specimen problem’, including sampling error (tumor cells were not aspirated) or a suboptimal specimen (‘scant but adequate sampling’ or ‘preparation artifact’), and ‘interpretation error’, meaning there were overdiagnoses or underdiagnoses by cytologists. All of the slides from the false-negative and false-positive FNAs were reexamined, and it was determined whether the misdiagnoses were due to a specimen problem or interpretation error.

### Statistical analysis

For “NIFTP=Ca” and “NIFTP≠Ca”, the sensitivity, specificity, positive predictive value (PPV), negative predictive value (NPV) and diagnostic accuracy were assessed.

Statistical analysis was performed using IBM SPSS Statistics (version 20.0). The variables were mainly categorical, and the chi-square test was used. A *P* value was considered significant when less than 0.05.

## Results

### Patients

Between April 2014 and March 2019, a total of 2781 thyroid FNACs were reviewed in our institution. Of the 2781 patients, 2109 were female (75.8%), and 672 were male (24.2%). The age range was 15–89 years. For the total 2781 FNACs, the incidences of the six diagnostic categories (I-VI) were as follows: 14.8, 17.1, 15.8, 2.3, 11.6, and 38.5%, respectively.

### Postsurgery histological findings of follow-up cases

In the entire study population, 1122 cases had available histological correlation data, of which 132 cases (11.8%) had nonneoplastic lesions, 17 cases (1.5%) had benign neoplasms, 969 cases (86.4%) had malignant neoplasms, and 4 cases (0.4%) had NIFTP. The FNA results were compared with the corresponding histological diagnoses (Table 1).

**Table 1:** The distribution of the cytologic-histologic correlation of the nodules

DC	Cases	non-neoplastic lesions	Neoplastic lesions							
			Benign neoplasms	NIFTP	Malignant neoplasms					
					Total	PTC	MTC	FTC	ATC	ITTA
I	42(10.2%)	17	0	1	24	24				
II	36(7.6%)	20	1	1	14	14			0	
III	90 (20.5%)	39	6		45	43	2			
IV	29 (45.3%)	12	7	1	9	8		1		
V	200 (62.7%)	41	2		157	149	7	1		
VI	725 (67.6%)	3	1	1	720	710	7		2	1
Total	1122 (40.3%)	132	17	4	969	948	16	2	2	1

*DC* Diagnostic category, *NIFTP* Non-invasive follicular thyroid neoplasm with papillary-like nuclear features, *PTC* Papillary thyroid carcinoma, *MTC* Medullary thyroid carcinoma, *FTC* Follicular thyroid carcinoma, *ATC* Anaplastic thyroid carcinoma, *ITTA* Intrathyroidal thymic adenocarcinoma

### The diagnostic utility of TBSRTC

For “NIFTP=Ca”, when DC V and DC VI both indicated positive findings (both need surgical excision according to the American Thyroid Association guidelines [8]), the sensitivity, specificity, PPV, NPV and diagnostic accuracy were 98.3, 30.9, 94.9, 58.3 and 93.5%, respectively. If DC VI was considered cytologic-positive, the sensitivity, specificity, PPV, NPV, and diagnostic accuracy of FNAC were 98.0, 84.0, 99.4, 58.3, and 97.5%, respectively.

For “NIFTP≠Ca”, when DC V and DC VI were both considered positive findings, the sensitivity, specificity, PPV, NPV and diagnostic accuracy were 98.4, 31.4, 94.8, 61.1 and 93.5%, respectively. If DC VI was considered cytologic-positive, the sensitivity, specificity, PPV, NPV and diagnostic accuracy were 98.1, 81.5, 99.3, 61.1, and 97.5%, respectively.

NIFTP does not affect diagnostic accuracy or notably influence the distribution of diagnostic errors.

### Analysis of the misdiagnosed cases

When DC V and DC VI were both considered cytologic-positive, there were 62 misdiagnosed cases, including 15 false-negative and 47 false-positive cases. However, if DC VI was considered cytologic-positive, there were only 19 misdiagnosed cases, including 15 false-negative and 4 false-positive cases.

We performed a retrospective review of the 62 false-negative and false-positive cases and found that 23 (38.1%) cases were due to specimen problem and 39 (61.9%) cases resulted from interpretation error. The 23 cases with specimen problems comprised 13 sampling errors and 10 suboptimal specimens. The main cause of the false-negative diagnoses was sampling error (13/15, 86.7%), while interpretation error led to most of the false-positive diagnoses (38/47, 80.9%). The analysis of the misdiagnosed cases is shown in Table 2.

**Table 2:** The analysis for the misdiagnostic cases

	Specimen problem		Interpretation error	Total
	Sampling error	Suboptional specimen		
False-negative diagnoses	13	1	1	15
False-positive diagnoses	0	9	38	47
Total	23		39	62

### Analysis of the false-negative cases

In a review of the 14 false-negative cases resulting from specimen problems, the findings demonstrated that the following factors may have contributed: papillary thyroid microcarcinoma (PTMC) (*n* = 6); thyroid cancer in patients with multinodular goiter (*n* = 4); diffuse fibrosis (*n* = 2); and nonspecific factors (*n* = 2).

Only one false-negative diagnosis was due to interpretation error; the postoperative histopathologic diagnosis of this case was follicular variant of papillary thyroid carcinomas (FVPTC), while cytopathologic evaluation result indicated a benign tumor. The analysis of the false-negative cases is presented in Table 3.

**Table 3:** The analysis for the false-negative cases

Analysis	Number
Specimen problem	
PTMC	6
Diffuse fibrosis	2
Thyroid cancer in patients with multinodular goiter	4
Nonspecific factors	2
Interpretation error	
FVPTC	1
Total	15

*PTMC* Papillary thyroid microcarcinoma, *FVPTC* follicular variant of papillary thyroid carcinomas

### Analysis of the false-positive cases

When DC V and DC VI were both considered cytologic-positive, 47 of our patients had false-positive diagnoses. Thirty-eight (80.9%) false-positive diagnoses were due to interpretation errors, and the postoperative histopathologic diagnoses of these cases were benign lesions with features mimicking or suggestive of a neoplasm including adenomatous hyperplasia (*n* = 17), thyroiditis (*n* = 12), and cystic lesions (*n* = 9). A suboptimal specimen is the second most common contributor to the false-positive rate. In this study, our data showed that six cases of thyroiditis, two cases of goiter with diffuse fibrosis and one case of PTMC were all misdiagnosed as PTC due to overlapping cytological features. The analysis of the false-positive cases is presented in Table 4.

**Table 4:** The analysis for the false-positive cases

Analysis	Number
Suboptional specimen	
Thyroiditis	6
Goiter with diffuse fibrosis	2
PTMC	1
Interpretation error	
Cystic lesions	9
Thyroiditis	12
Adenomatous hyperplasia	17
Total	47

*PTMC* Papillary thyroid microcarcinoma

Regarding positive findings for DC VI, there were a total of 4 false-positive diagnoses, including adenomatous hyperplasia (*n* = 2) and cystic lesions (*n* = 2).

## Discussion

### The diagnostic utility of TBSRTC

FNA is a simple, safe, cost-effective and accurate diagnostic tool for the initial screening of patients with thyroid nodules [8–10]. The aim of FNA is to identify neoplastic nodules for surgical removal while avoiding surgical intervention for nonneoplastic lesions. In our study, when DC V and DC VI were both considered cytologic-positive, the sensitivity, specificity, PPV, NPV and diagnostic accuracy were 98.3, 30.9, 94.9, 58.3 and 93.5%, respectively. If DC VI was considered cytologic-positive, the sensitivity, specificity, PPV, NPV and diagnostic accuracy of FNAC were 98.0, 84.0, 99.4, 58.3, and 97.5%. Therefore, a key point of our study is that DC V is not highly reliable for the diagnosis of malignancy, and when excluding this category, the diagnostic accuracy and especially the specificity, increase. Therefore, for cytological diagnoses of DC V, a frozen section analysis during diagnostic lobectomy is necessary. The PPV and diagnostic accuracy all exceeded 90.0%, indicating that thyroid FNA is an important part of the preoperative diagnosis.

It is well known that thyroid FNA derives much of its clinical value from its ability to reliably enable the identification of benign thyroid nodules and its low false-negative rate, which enables surgeons to use FNA as a reliable test for guiding operative decision-making; thus, the lower the false-negative rate is, the more valuable thyroid FNA will be. Each category of the TBSRTC has an implied cancer risk, which ranges from 0 to 3% for the “benign” category to virtually 100% for the “malignant” category [7]. The false-negative rates of FNA for thyroid nodules reported by most studies are less than 5% [11–16]. However, higher rates (varying from 7.5 to 21%) have also been published in other study series [17–22]. In the present study, the false-negative rate (1.7%) was lower than those of reported studies, demonstrating that our negative thyroid FNA results were fairly reliable. However, the relatively low NPV (58.3%) showed that despite a thyroid nodule being initially diagnosed as benign by FNA, it may have malignant potential. Moreover, it is noteworthy that the categorization of cases at DC I or DC II does not mean “negative for malignancy”, and these patients should undergo regular, close follow-up or diagnostic lobectomy if the clinician thinks it is possible that the tumor is malignant. We acknowledge that the NPV data are more informative because most cytologically benign thyroid nodules do not require surgery.

### Analysis of the false-negative cases

In our study, most false-negative diagnoses were caused by specimen problems (87.5%, 14/16), and 6 of 14 cases had PTMC with a resultant sampling of adjacent normal tissue. Papillary thyroid microcarcinoma (PTMC) is defined by the World Health Organization (WHO) as a PTC whose longest diameter measures ≤1.0 cm and that is found incidentally [23]. As previous studies had noted, PTCs measuring less than 1 cm in diameter are usually incidental and discounted as false-negative by some pathologists [24–27]. In fact, in some studies, the most common cause of a false-negative diagnosis by FNA is the presence of an unsampled microcarcinoma in the setting of an adenomatous goiter [15, 28]. Notably, thyroids with large nodules may harbor microcarcinomas within the nodule; moreover, once a nodule reaches a certain size, it may be difficult to precisely sample the entire nodule [29], and an increased false-negative rate for larger nodules has been reported by some authors [27, 30–33]. Several researchers have reported false-negative rates as high as 17 to 19.3% for thyroid nodules ≥3 to 4 cm [19, 20]. However, this concept had been challenged by other reports showing that a large nodule size neither diminishes the accuracy of FNA nor increases the risk of malignancy within the aspirated nodule [34, 35]. We support the former viewpoint for the following two reasons: first, nodule heterogeneity has been revealed to be one of the underlying causes of misdiagnoses, which may explain the difficulties in evaluating these nodules by traditional cytologic methods [32], and second, it should be noted that the number of patients undergoing surgery in the latter series with the opposing view (*n* = 145 and *n* = 127) may not have been sufficient to demonstrate the significant effect of nodule size, even if one existed.

Another factor of great concern regarding false-negative diagnoses is the number of nodules, and sampling error may occur when the cells captured by the needle are not from the targeted nodule. On the basis of reviewing the 4 false-negative cases with multiple nodules in our study, we found that a benign nodule was located preoperatively, while the hidden nodule in the tumor was missed. Studies have demonstrated the high incidence of malignancy in patients with multinodular goiter compared to the general population [36] and that the presence of multiple nodules can hamper the evaluation of the entire thyroid [37]. Therefore, false-negative results may arise due to heterogeneity within the targeted nodule or among diverse nodules, so multiple passes should be performed in various parts of a large nodule or from different nodules to reduce the risk of false-negative findings owing to heterogeneity.

Our data suggest that interpretation error was not the major cause of false-negative diagnoses (*n* = 1). We found only one case of a FVPTC that was classified as benign by cytopathologic evaluation. Previous authors have also noted FVPTC to be an important factor in false-negative thyroid FNA results, and the value of thyroid FNA is limited by its inability to distinguish follicular lesions reliably [16, 38–41]. Histopathologic features of FVPTC tend to overlap with those of follicular neoplasms, hyperplastic adenomatoid nodules in goiter or even lymphocytic thyroiditis, as the characteristic nuclear features often present with subtle nuclear changes. In addition, nodule heterogeneity is also a particular problem with FNA of FVPTC, whose histological diagnoses of malignancy may be made in accordance with nuclear features of PTC while the features present only focally in the nodule. As a result, this tumor is frequently misinterpreted as a follicular neoplasm or an adenomatoid nodule. We acknowledge that for these follicular lesions, cytopathology is a screening tool rather than a diagnostic test and that the main goal of cytopathology is not to establish an absolute diagnosis but to determine the correct management for the patient. Şule Canberk et al. [6] have also emphasized that remembering three “As” could prevent misinterpretations and enable better clinical management: be aware of the limits of cytomorphology; be awake to the presence of sheets/macrofollicles, abundant colloid, lymphocytes, and obscuring blood; and avoid down-grading nuclear atypia.

### Analysis of the false-positive cases

The 47 thyroid FNACs with a histopathology-proven false-positive diagnosis demonstrated that the following were the major contributing factors to false-positive cytologic diagnoses: adenomatous hyperplasia, thyroiditis and cystic lesions.

Our data suggested that hyperplastic and adenomatoid nodules represented the most significant pitfall in thyroid FNAs, in which these types of pathology can be misinterpreted as suspicious for PTC or PTC. Benign thyroid hyperplastic nodules typically show follicular epithelial cells with small, round, dark nuclei in a honeycomb arrangement. However, focal nuclear atypia that can be mistaken for PTC, including grooves, an oval shape, chromatin clearing, and overlapping, have been reported in hyperplastic nodules, which leads to diagnostic difficulties [42, 43]. It is worth mentioning that benign thyroid nodules with papillary hyperplasia can pose a diagnostic challenge not only in cytology but also in surgical pathology by mimicking classical PTC [44–47]. Marc P et al. [42] have underscored that benign thyroid nodules with papillary hyperplasia should be considered when a FNA reveals papillary structures with sparse nuclear features of PTC or features that are mixed with an otherwise benign-appearing follicular component. Other researchers have stressed that nuclear overlapping and crowding is a rare finding in specimens of benign thyroid hyperplasia and that the presence of abundant loose or watery colloid can prevent overdiagnoses by cytopathologists [48, 49].

Previous studies have demonstrated that thyroiditis is one of the most common factors in the false-positive diagnosis of PTC [28, 43, 50–52]. We believe that the serious overlap in morphological features between PTC in chronic lymphocytic thyroiditis and pure thyroiditis can pose a challenge for the cytopathologist, and even an experienced cytopathologist may be uncertain about the diagnosis between these two entities. In our experience, 18 of the 47 false-positive cases were thyroiditis, including 9 cases of granulomatous thyroiditis and 4 cases of Hashimoto’s thyroiditis. Diagnostic traps in the cytological evaluation of thyroiditis vary depending on the stage of the disease. The ‘cellular stage’ is composed of a proliferation of oncocytes, and an oncocytic change may lead to some nuclear atypia: nuclear enlargement, fine chromatin texture, prominent nuclear membranes and macronucleoli. Occasional nuclear grooves or pseudoinclusions and a paucity of background lymphocytes can lead to overdiagnoses of PTC. In contrast, the ‘fibrotic stage’ is widely fibrotic, and sclerosing and may yield few cells upon FNA. However, squamous metaplasia of thyroid follicular epithelial cells may be misinterpreted as suspicious for malignancy. In such cases, scanty cellularity can be considered worrisome in the case of the presence of some atypical cells suggesting PTC [6, 53, 54]. There are some key diagnostic clues that can help distinguish between reactive nuclear changes and PTC in chronic lymphocytic thyroiditis. The reactive follicular epithelium in chronic lymphocytic thyroiditis is usually adjacent to the inflammatory infiltrate and with focal nuclear atypia showing some but not all features of PTC [53, 55]. Identifying the multinucleated giant cells, epithelioid histiocytes, and fibrotic stromal fragments in the complex background created by acute-chronic inflammation and debris can improve the sensitivity for the diagnosis of granulomatous thyroiditis [56–58]. However, it should be noted that the presence of multinucleated giant cells does not rule out the diagnosis of thyroid cancer. The diagnosis of granuloma must be based on careful observation of all sections and the absence of thyroid cancer cells.

In evaluations of cystic nodules, the role of FNAC is limited, and false-negative diagnoses of malignant cysts are well documented [20, 59–63]. However, only a few authors have emphasized that atypical cyst-lining cells may produce false-positive cytologic results, and few have fully demonstrated the cytologic and histologic features of atypical cyst-lining cells [5, 61]. Our study found that 9 cases of benign cystic lesions were misdiagnosed as suspicious for PTC, and we retrospectively scrutinized the smears. Consistent with the opinion of Faquin et al. [61], the “atypical” cells demonstrated a cytomorphologic spectrum from spindle, elongated cells to polygonal, epithelioid cells with nuclear enlargement, nuclear grooves, fine chromatin, and distinct nucleoli while lacking nuclear crowding, such as intranuclear pseudoinclusions, and the papillary architecture of cystic papillary carcinomas. Malheiros et al. [5] have also emphasized that if atypical features are seen in smears from those cystic lesions with a paucicellular background, the diagnosis of PTC should be made with great caution only if we find unequivocally nuclear features of PTC. Accordingly, we recommend that accurate identification of atypical cyst-lining cells and their background is crucial to distinguish the benign nature of cystic thyroid nodules and avoid unnecessary operations.

### Intraoperative frozen examination in misdiagnosed cases

For DC I, DC II and indeterminant cytologic diagnoses, if the clinician still suspects malignancy, intraoperative frozen examination is helpful to determine the nature and scope of the operation. At our institution, 54 of the 62 misdiagnosed cases had frozen sections; among those cases, 11 (20.4%) had inconclusive frozen section results. It is important to note that among the false-positive cases, 5 cases were suspicious for PTC, and one case was diagnosed as PTC based on the frozen section analysis. Massimo et al. [64] explained that there are two main limitations for frozen section analysis. First, the quality of the specimen obtained for a frozen section is lower than that obtained during routine histopathological examination. Second, the single frozen section obtained may not be representative of the lesion as a whole. Thus, we must acknowledge that it is difficult to achieve an accurate diagnosis by FNAC for some cases.

## Conclusions

The sensitivity and PPV of thyroid FNAC in our institution were higher than those of other institutions, while the specificity and NPV were lower. Specimen problems were the main causes of false-negative diagnoses, while interpretation error led to most of the false-positive diagnoses. Multiple passes should be performed in various parts of a large nodule or in different nodules to reduce the false-negative rate due to specimen problems. Cytopathologists should strengthen their criteria for the identification of adenomatous hyperplasia, thyroiditis and cystic lesions to avoid false-positive diagnoses due to interpretation errors. NIFTP does not affect the diagnostic accuracy or notable influence the distribution of diagnostic errors.

## Data Availability

The datasets used and/or analyzed during the current study are available from the corresponding author on reasonable request.

## Abbreviations

AUS/FLUS: Atypia of undetermined significance or follicular lesion of undetermined significance
DC: Diagnostic categories
FNA: Fine-needle aspiration
FNAC: Thyroid fine-needle aspiration cytology
ND/UNS: Nondiagnostic or Unsatisfactory
NIFTP: Non-invasive follicular thyroid neoplasm with papillary-like nuclear features
NPV: Negative predictive value
PPV: Positive predictive value
PTC: Papillary thyroid carcinoma
PTMC: Papillary thyroid microcarcinoma
SFN/FN: Suspicious for follicular neoplasm or Follicular neoplasm
SM: Suspicious for malignancy
TBSRTC: The Bethesda System for Reporting Thyroid Cytology
